# Effects of a Fragmented View of One’s Partner on Interpersonal Coordination in Dance

**DOI:** 10.3389/fpsyg.2016.00614

**Published:** 2016-05-02

**Authors:** Derrick D. Brown, Ruud G. J. Meulenbroek

**Affiliations:** Donders Institute for Brain, Cognition and Behavior – Donders Centre for Cognition, Radboud University NijmegenNijmegen, Netherlands

**Keywords:** dance cognition, interpersonal coordination, joint action, kinematics, motor control, performance science

## Abstract

In this study we investigated the effects of a mirror-mediated, partial view of one’s dance partner on interpersonal coordination in dance duets. Fourteen participant pairs (dyads) were asked to perform a reflectionally-symmetric eight-segment dance-relevant arm movement sequence in two visual conditions: with one dancer facing the mirror and providing a partial view on the dance partner, or both dancers facing back to back with, for both dancers, no view on one’s partner. During an eight-count beat-preparation phase, the task was paced via a metronome at three TEMPI; 1.6, 1.9, and 2.3 Hz, which was subsequently removed after which the movement sequence continued in silence. Interpersonal coordination was assessed using two tri-axial wireless accelerometers, one fixed to each dancer, that allowed the off-line kinematic analyses of dyad correlation, mean relative phase and mean standard deviation of relative phase of the up–down movements of (one of) the hands of the two dancers. In addition, two independent raters estimated the realized movement frequencies and percentage of the trial duration that the dancers moved in sync. Repeated measure ANOVAs revealed systematic effects of tempo on the performance measures, a positive effect of the use of the mirror on the coordination of the dancers’ movements but no facilitating effect of the mirror on the dancers’ synchronization. Overall, the results support the contention that when dancing to an internalized rhythmic beat the use of a mirror provides an ecological means to stabilize interpersonal coordination in dance duets without an effect on synchronization.

## Introduction

### Dance

Dancing is a complex task involving a plethora of motoric, cognitive, and affective processes. Dancing together with two or more individuals at the same time exemplifies such complexity; as the information processes that afford solo dance need to be properly coordinated between individuals. Apart from being dependent on techniques and artistry, successful joint dance may depend on anticipatory information processing in sensory, motor, and cognitive modalities. For example, dancers exhibit a better integration of proprioceptive signals than non-dancers when given an endpoint position-matching task (Jola et al., 2011). When compared with non-dancers, trained dancers are more successful at visual-motor coordination of dance moves (Washburn et al., 2014). Thus being able to form proper mental representations of dance movements may be an essential prerequisite for learning dance (Bläsing et al., 2009; Bläsing and Schack, 2012). Knowing how to adapt movements in real-time and improvise in movement, is another skill crucial to flexibly generate unplanned, new dance patterns. In dance genres containing specific techniques, e.g., Cunningham, ballet, and Graham, movement sequences are planned based on syllabi, which make up a movement repertoire. In such structured dance forms, pre-planned movements may depend on the goal and the position or posture of the body at the start of movement execution, a process which has been researched at both the behavioral and neural levels (Rosenbaum et al., 1999; Zimmermann et al., 2011). In dancing together, such goal-posture planning might be relevant as choreographed dance seems to rely heavily on the shared representation of the rehearsed (planned) action goals (Decety and Sommerville, 2003; Newman-Norlund et al., 2007). The central goal of the present study is to investigate the effects of the use of a mirror on interpersonal coordination in dance duets who perform preplanned movement sequences together at the same time, i.e., whether or not mirror-use facilitates interpersonal rhythmic coordination. To start, we extend our queries within the precepts of joint action and then we will highlight the sensory modalities of audition and vision and their relationship to dance.

### Synchronization in Joint Action and Joint Coordination

Humans are experts in collaboration when trying to accomplish tasks they cannot achieve alone (Bekkering et al., 2009). It has been suggested that real-world (non-lab) joint-action tasks depict the ability to take into account a partner’s potential action capabilities as they relate to the goal of a particular task (Obhi and Sebanz, 2011). Previous research in joint-action studies range from observing the continuous joint involvement of lifting and balancing an object (Meulenbroek et al., 2007; Newman-Norlund et al., 2007), to musicians’ temporal coordination in a music performance (Loehr and Palmer, 2011). Audition and vision play a central role in facilitating joint-action coordination (Schmidt et al., 1990; Schmidt et al., 2007). Moreover, when learning dance, visual stimuli via the mirror mediate error detection and movement-sequence correction (Brodie and Lobel, 2008). Consequently, audition and vision will be controlled in our study on the effects of the use of the mirror on dyadic coordination during a dance sequence. In dance duets and ensembles, success depends on many factors such as knowledge of one partner’s intentions, interpretation of verbal instructions provided, anticipation of unrehearsed movements, musical or rhythmic alignment, correction of errors within and between partners, skill differences, and depending on the genre of dance, anthropometric constraints (e.g., combination of a tall and short dancers). Dance dyads daily employ concepts of joint coordination, joint observation, and joint imitation since dance, as a profession, is joint action.

### Audition

Apart from organizing movement through physical contact, hearing, perceiving, and consequently acting on an external beat or pulse is integral to dance. Tempo in dance can be defined as the rate at which repetitive movement occurs (Moelants, 2002) and embodied in how one perceives the beat. Tempo is normally set by the instructor and often depends on the type of movement that needs to be executed. In classical dance for example, in order to prepare dancers to coordinate tempo and style quality with the movement they are learning, instructors use terms synonymous with music; adagio and allegro; slowly and lively or 1.2 and 2.3 Hz, respectively. Dancers then hear a preparation, usually a four count or eight count preparation played by the pianist to introduce both the melody and tempo he or she will play proceeded by the start of the dance phrased introduced by the instructor. Beat induction is proposed as a sensory guided action represented at the musculoskeletal (McAngus Todd et al., 1999) and cognitive levels (Honing, 2012), that allows the hearing of an ordered musical pulse. So the perceptual modality of rhythm, within a musical phrase “locks” agents into forming dyadic unison. In Western style dance classes beat induction may occur with the aid of a musician as mentioned above or recorded music accompanying the dance (Sofianidis et al., 2012). Beat induction can also occur via a dance instructor, as a voiced eight count call-out, followed by silence as dancers attempt to internalize the external beat just heard. This would be seen in a contemporary style dance class for example. In contrast, in Non-Western dance genres and in choreographic performance, beat induction need not be present. Dancers and choreographers may seek a variety of ways to coordinate with or without rhythm or metered regularity (Vass-Rhee, 2010). They may exploit breathing cues, ambient sounds, or attend to mono or polysyllabic utterances to organize choreographies with fellow performers. With regard to audition and moving together, there is extensive research that reveals rhythmic joint-action coupling via timing and affect in musicians and dancers (Sevdalis and Keller, 2011; Keller et al., 2014). Scholars have captured sensorimotor coordination modes in urban dancers via an externally provided rhythm matched with corresponding movement (Miura et al., 2013).

### Vision

Regarding spurious interpersonal coordination on the basis of vision, two persons spontaneously swaying side by side when sitting in rocking chairs (Richardson et al., 2007) is a convincing example. Bosga et al. (2010) highlighted the importance of online visual feedback with a partner when jointly balancing on a curved surface. Fine and Amazeen (2011) revealed spontaneous coordination for both intrapersonal and interpersonal coordination in a rhythmic tapping task due in part to visual coupling. In dance, vision unlike audition, requires a more extensive characterization.

A distinct feature that separates dance from other professions is the use of mirrors. In our daily life, mirrors afford us rich ways to interact with the world around us. Even in seemingly dangerous situations such as driving in traffic while applying make-up, mirrors give many the confidences to conduct both tasks simultaneously with relative ease. Such multitasking via the mirror may require simultaneous integration of information, or multiple task constraints as it were. In behavioral studies observing infants, mirrors convey a particular type of visual perception in self-recognition (Gibson, 1969), thus coordination of limb movements may be improved by utilizing the mirror. Mirrors might also be used to inform perceptual learning (Loveland, 2003) for a mirrored surface placed in an environment, can afford the perceiver a tool for action. In addition, the spatial position of body parts reflected in the mirror might be relevant for self-recognition (van den Bos and Jeannerod, 2002) and for imitating one’s own movement, alluding to links between sensory and motor representations (Heyes, 2001). Further, our ability to recognize ourselves while creating movements in the mirror may depend, at least partly, on the plasticity of the representation of our own body and peripersonal space (Holmes et al., 2004). In dance, particularly duets, this equates to a continual mirror referencing and updating of collective gestures or kinesics, and the kinaesthetics imparted in learning dance techniques and eventually performing planned (i.e., choreographic) sequences onstage. It is relevant to note this *mirror-mirroring* does not often happen on-stage. Embedded in dance training via mirror-mirroring is a cognizance of bodies co-joined in action in a 2-D and 3-D environment.

Historically, it is not clear when the first mirrors were placed in a dance studio. King Louis XIV, seen as the first royal patron of ballet as we know it today, was himself an avid dancer. His palace at Versailles contains The Hall of Mirrors which might have served as the first mirrored dance studio. Pedagogically, mirror use is not explicitly codified in any of the classic ballet, modern or contemporary techniques as a referential instrument for instruction. Nevertheless its presence has become ubiquitous in how dancers learn to dance. Once a pre-professional dance student starts to train he or she will spend on average 5 h a day, ±1500 h a year using the mirror to learn the different styles of dance and choreographies they will eventually perform. This continues throughout the subsequent two or more decades that a dancers’ profession entails. For a classical ballet student, completing the standard 10-year education starting from age 9, this is a markedly large time devoted to seeing oneself and others via the mirror. Via an 8–10 m wall lined with mirrors, dancers interact with themselves and others via mirror images and bodies in live space. At a direct level of action and perception, when facing a mirror, we encounter ourselves as we see and are seen by others; namely our reflection is left right reversed. In the dance studio, one’s full-body image reflection is often obstructed, and so a duet or ensemble can be seen rehearsing a dance in unison while only partially observing themselves and confederates in the mirror. An ecological example is a large dance audition for a conservatoire or company. In this instance, there are multiple environmental constraints to contend with. Forty or fifty dancers may “compete” in peripersonal and extrapersonal space, to move unhindered, see clearly movement sequences provided by one or two instructors and do so without being hit by flying limbs. In real time, dancers, while watching the instructors, attempt to “visually lock” to a fragmented view of their own and colleague’s 2-D image while also dancing in their 3-D shared workspace. Remarkably, the instructor can either face the mirror or face the dancers while providing the movement adding to the cognitive complexity of processing the task given. Moreover, the self/other image presented to dancers is occluded and fragmented as reflections of the body from the waist down are often incomplete due to the row of tables placed exactly in front of the mirrors while the auditors observe the auditionees and make their selection. Visuo-cognitively, this raises questions as to how dancers orient action within a shared workspace and effectively process the critical information needed to dance together without error-based collisions. There is scant published literature that examines specifically the use of the mirror- as a tool for dancing with a partner. However, dance education researchers have highlighted mirror use with regard to body image challenges (Radell et al., 2011; Radell, 2012) while others have used the mirror as a tool for teaching balance in solo young dancers (Notarnicola et al., 2014). Moreover, it has also been suggested that an overreliance on reflected visual stimuli may diminish the much needed multisensory attention to action (Brodie and Lobel, 2008) and other perceptual modalities necessary in dance (Kasai and Parsons, 2003).

In sum, above we provided evidence from studies that reveal the use of audition and vision as modi for individual as well as joint coordination. In the present study, we investigate the effects of the mirror and beat induced TEMPI on motor coordination across individuals in a dance duet. We contend that *mirror mirroring* of self and others’ reflection must be calibrated in novel ways to form a cohesive reproduction of what eventually is danced together. A rather obvious yet empirically underexplored question is whether the mirror affords dyads or ensemble dancer’s successful coordination when exploiting this tool. It was hypothesized that pre-professional dancers will coordinate their movement sequences when at least one partner of the dyad faced the mirror independent of an instructed but a self-maintained tempo. Further, we predicted that the mirror would facilitate dyad coordination independent of dance tempo.

## Materials and Methods

### Subjects

Fourteen (*N* = 14, 3 male, 11 female; mean age 19.71 years ± SD 2.09 years) pre-professional dance students from the ArtEZ Institute of the Arts (Arnhem, the Netherlands) bachelor of dance program volunteered to participate in the study. Twelve participants were right-handed and two were left-handed. Handedness was determined using a modified version of the Edinburgh Handedness Inventory (Oldfield, 1971). Participants were first-year students all with at least 5 years of dance training prior to attending the current institution. The Radboud University’s Institutional Review Board approved the experiment. All participants provided informed consent, completed EHI forms and were subsequently randomized into dyads.

### Procedure

**FIGURE 1:**
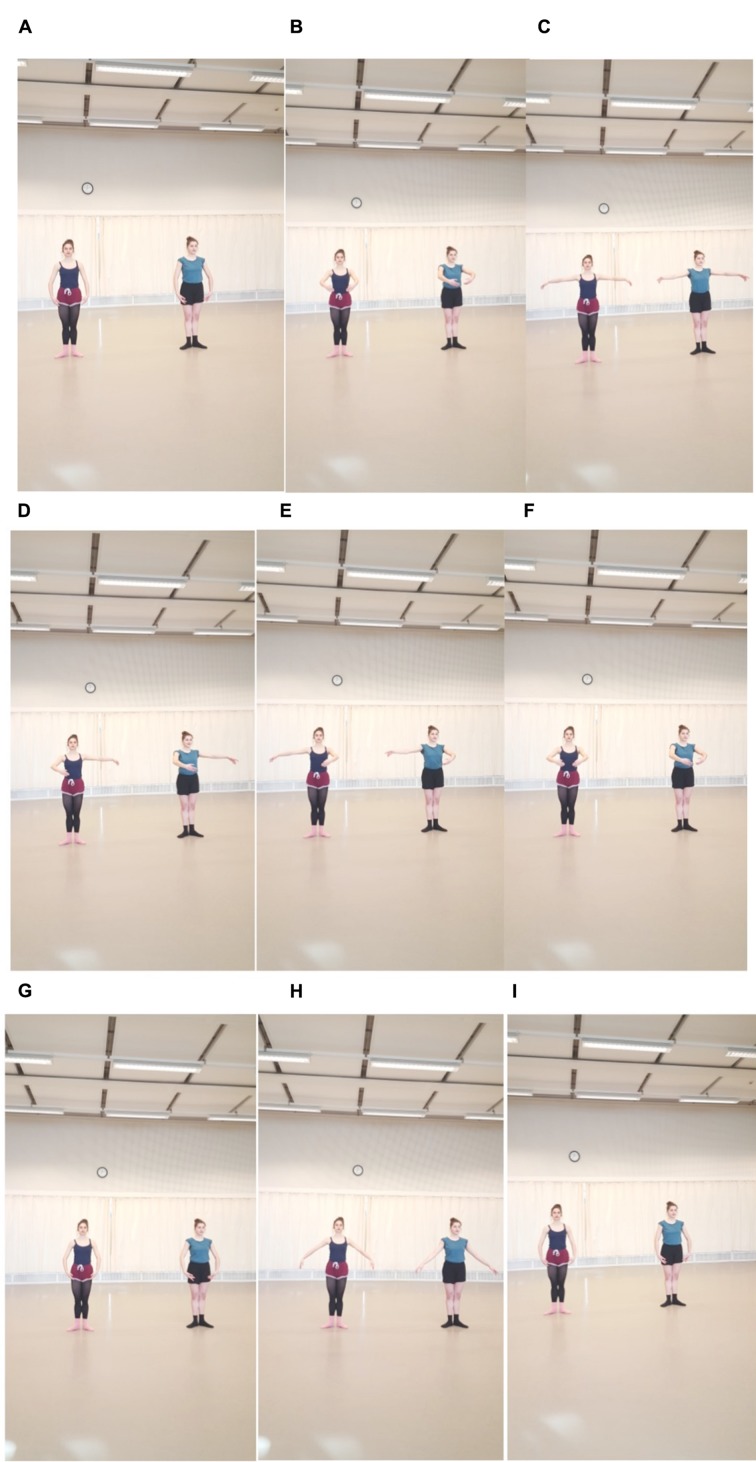
**Example movements from Sequence A–I; *bras-bas*, 1st, 2nd^,^ 3rd, 3rd reversed, 1st, *bras-bas, demi seconde allongé and bras- bas***.

Dance training traditionally occurs in a dance studio, thus the current study exploited this ecological feature, by conducting this investigation in the dancers’ natural environment. The protocol comprised a dance sequence classified as low in intensity, thus participants required no special warm-up, and however, they were requested to dress in their normal dance attire. The curtains, utilized in a dance studio were closed in a manner that allowed the dancers to see only a 1 m in diameter self-reflection and only fragmented reflection of the confederate’s body in the mirror. The dancer facing the mirror stood spatially anterior to the dancer facing the back wall; facing downstage versus upstage respectively in theater nomenclature. Thus for the dancer facing forward, when viewed along the coronal and sagittal planes, the most lateral side of the confederates body was occluded. Each dyad was asked to stand in the center of the dance studio approximately 1 m apart and in the center of the room approximately 4 m from the mirror. To control for a ‘rehearsal bias,’ where dancers first practice together the task required, only one dyad was allowed in the studio at a time and the randomization of dyads was only known to participants once the principal investigator announced names of the next dyad to enter the studio. The arm positions were familiar to pre-professional dancers as they are practiced in multiple combinations on a daily basis. The order, however, was new and was provided by the researchers verbally before the start of the trials so as prevent visual imitation of movement. In classical dance terms, the participants completed on *port de bras* arm sequence using eight positions: seen in **Figures 1A–I**, in classical ballet lexicon as; *port de bras* in *bras-bas*, 1st, 2nd, 3rd, 3rd reversed, 1st, *bras-bas, demi seconde allongé and bras- bas*, respectively. We informed the participants they would hear an eight-count beat provided by a digital metronome that was their preparation. After the eight-counts they were instructed that the beat would be removed and they were simply to continue in the same tempo dancing the arm phrase together at the same time in three different positions; in dance terms one dancer facing upstage the other downstage and back to back, with no further instructions given. There was no additional coaching provided by the researcher present. Once the instructions were clear, each participant of the dyad faced each other and completed two rounds of the sequence at 1.6 and 2.3 Hz, which served as the baseline. These recordings were not included in the final analysis.

### Design

**FIGURE 2:**
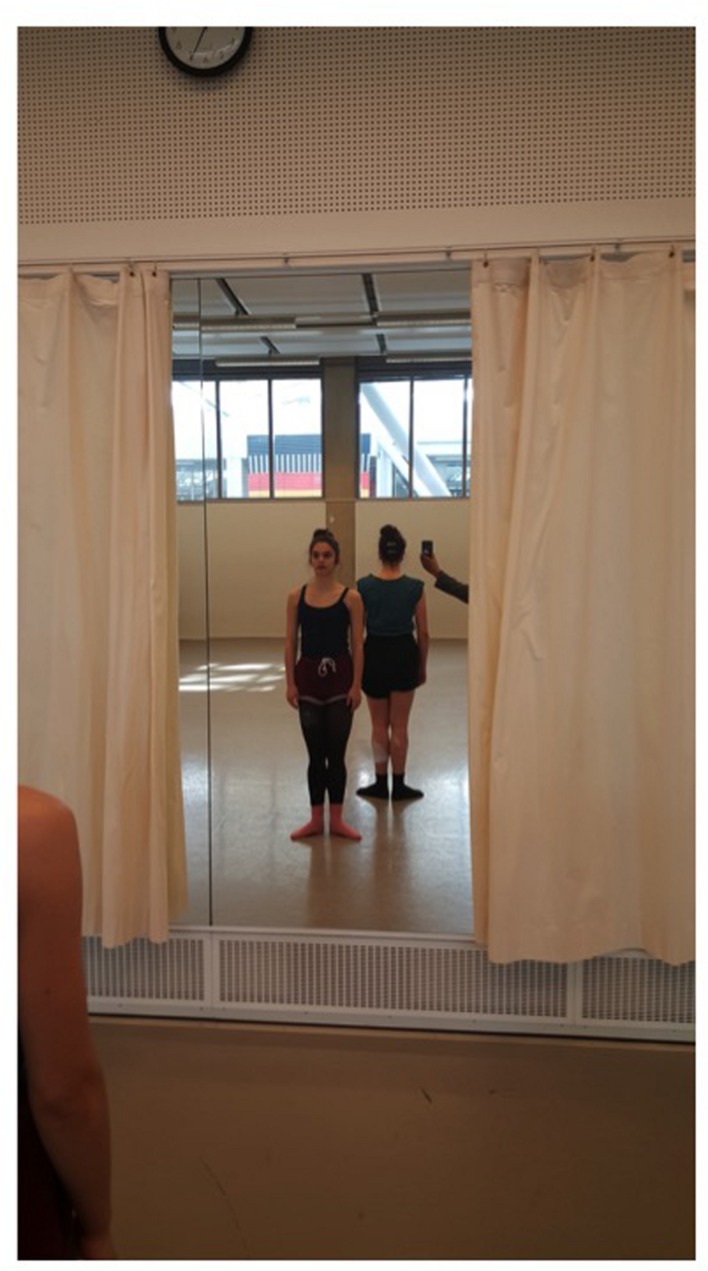
**Facing mirror (FM)**.

Participants were asked to dance together one continuous arm sequence that consisted of eight arm positions moving along the transverse-sagittal planes. From a motor behavior perspective the arm phrase would be considered *reflectionally* symmetric within the dyad; that is the participants performed a physically identical and identically oriented movement phrase (Amazeen et al., 1998; Brick and Boker, 2011). To test the effects of the use of the mirror (VIS) on the dyads’ coordination, the participants completed the arm sequence under two conditions: (1) FM: one dancer within the dyad facing the mirror while the other dancer faced the back wall as shown in **Figure 2**, and, as a control condition: (2) NM: no mirror use, i.e., the two dancers facing back to back with a meter between them, both without visual access to the mirror. The FM condition was counterbalanced across the two dancers: in half the trials Dancer 1 faced the mirror, in the other half Dancer 2 faced the mirror.

To test for auditory-lock (TEMPI), dancers were prompted with a sinusoidal acoustic stimulus heard for eight counts, which immediately switched off at the moment the dancers commenced with performing the movement sequences. The sinusoidal signal was set to three dance-practice relevant frequencies in random order: 1.6, 1.9, and 2.3 Hz, in music terminology, equivalent to *Andante*, *Moderato*, and *Allegro*, respectively. Each dyad made a total of 48 counterbalanced matching attempts: 2 COND × 3 TEMPI × 8 REPETITIONS.

### Materials

The movement data from each dyad was recorded using two tri-axial wireless accelerometers (KinetiSense^TM^ 6-Axis Motion Sensors). One accelerometer was placed in the posterior direction on the right wrist of each participant. The axes of the unit aligned with the body in standard anatomical positions: the *x*-axis was aligned with the frontal plane, the *y*-axis on the transverse and the *z*-axis on the sagittal plane. The command module was attached laterally or anteriorly (via clip) for ease on one of the participants. The shared workspace was maximum 1 m between participants.

### Data Analysis

**FIGURE 3:**
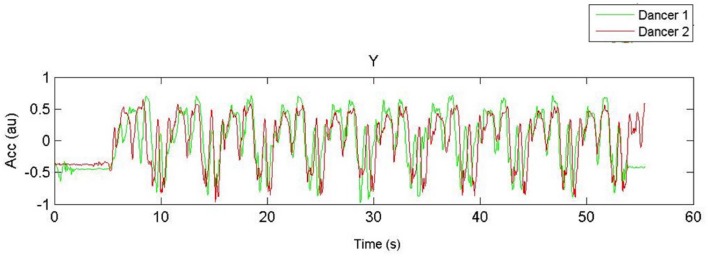
**Acceleration-time function of the hand movements of Couple 3, facing away (NM) 1.6 Hz**.

**FIGURE 4:**
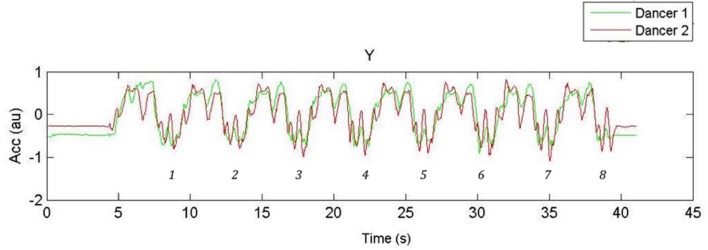
**Acceleration-time function of the hand movements of Couple 1; 1 dancer facing the mirror (FM) 1.9 Hz with 8 repetitions of movement sequence demarcated**.

Dyad coordination was calculated using the raw accelerometers signals of the *y*-axis. Dyad correlations, the continuous relative phase, and the standard deviation of the continuous relative phase of the vertical (up–down) right-hand accelerations were calculated after applying a Butterworth low-pass filter with cut-off frequency of 5 Hz. The accelerometer signals (input noise <60 mg RMS) were linked wirelessly (2.4 GHz radio) to the laptop and sampled at 128 Hz. Data acquisition was conducted using the software program SoapSynergy^®^. Raw data images from all trials were visually inspected after each trial to ensure actual signals from accelerometers and sinusoidal signal were properly captured throughout the trial. In addition, the raw data plots were rated off-line by the first author (DB) and one independent rater who was naïve as regards the experimental conditions, to derive quantitative estimates of the realized frequency by each dancer per trial and their synchronization (PercTsync). PercTsync was thus not based on a computer algorithm but based on visual inspection of the raw data plots for each trial on a separate page, showing the position traces of the two actors. **Figures 3** and **4** show two examples of such plots. Given that each arm sequence consisted of 8 movements/positions, the participants realized 8 × 8 = 64 movements provided they completed the entire sequence. For each trace the rater was asked to note (a) how many segments each dancer completed, (b) at which point in time (in second) each dancer started the sequence, (c) at which point in time (in second) each dancer finished the movement sequence, and (d) at which point in time (in second) the two dancers went out-of-sync. Subsequent analyses were conducted in Matlab^®^ R2012b (Mathworks).

### Statistical Analysis

Four repeated measure ANOVAs were applied for the movement correlations, the mean relative phase, and the standard deviation of the relative phase, and percentage of time dancers were in synchrony (PercTSync) separately. Sphericity of the data was assessed using Mauchly’s test with α set to 0.05. In the case of a positive Mauchly’s test, a Greenhouse–Geisser correction was conducted during the analysis to generate accurate α scores. SPSS 22.0 was used for statistical analysis.

## Results

### Interclass Correlation Coefficient

The interclass correlation coefficient was calculated to assess the agreement amongst the two raters, which for the realized frequency was ICC = 0.92 indicating a strong inter-rater reliability. The realized frequencies at the slow (1.6 Hz), moderate (1.9 Hz) and high TEMPI (2.3 Hz) were 1.62, 1.72, 1.97 Hz, respectively, showing that the lowest tempo was maintained correctly across the trial duration whereas the medium and fast tempo were not achieved.

### Correlation between Vertical Hand Accelerations

**FIGURE 5:**
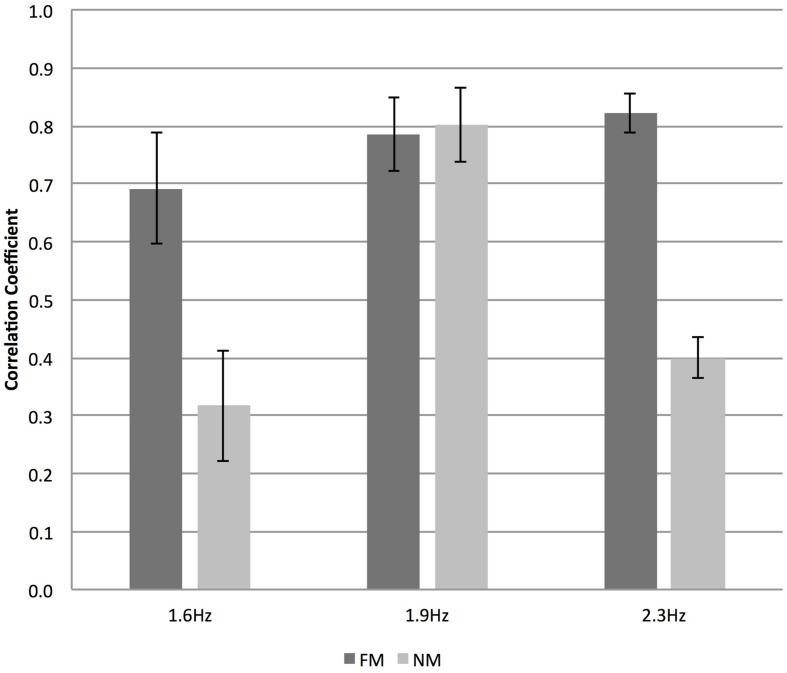
**correlation of the vertical hand acceleration profiles in dyads as a function of COND (FM versus NM) and TEMPO (Hz).** Error bars denote between-subject (dyad) standard error.

**Figure 3** shows a 60-s time window plot of the vertical acceleration patterns of the two hands of the dancers performing at 1.6 Hz in the NM-condition. **Figure 4** shows a similar example of hand-acceleration patterns recorded at 1.9 Hz in the FM-condition in which the eight repetitions of the movement sequences can be clearly discerned. With regard to movement coordination, there was a statistically significant effect of VIS on dyad coordination as captured by the correlation of their vertical hand accelerations, *F*(1,6) = 90.831, *p* < 0.001, $\eta_{p}^{2}$ = 0.938 showing that dyads were successful at coordinating when either partners faced the mirror (see **Figure 5**). With regard to TEMPI there was a statistically significant effect on the correlation between the dyad’s vertical hand accelerations, *F*(2,12) = 6.134, *p* < 0.015, $\eta_{p}^{2}$ = 0.506, which, on average, proved the highest in the 1.9 Hz tempo. Further, the ANOVA yielded a significant interaction between VIS and TEMPI on the correlation of their vertical hand movements, *F*(2,12) = 6.395, *p* < 0.013, $\eta_{p}^{2}$ = 0.516. A series of LSD corrected pairwise comparisons revealed a significant difference in coordination at TEMPI 1.6 Hz (*p* = 0.002) and 2.3 Hz (*p* = 0.006) when our dyads either faced the mirror (FM) or danced back-to-back (NM). However, at tempo 1.9 Hz VIS had no significant effect on coordination within the dyads (*p* = 0.841).

### Mean Relative Phase

For the mean relative phase, the ANOVA yielded no systematic variation of in-phase coordination for the main conditions COND, *F*(1,6) = 1.449, *p* > 0.05, TEMPO, *F*(2,12) = 0.584, *p* > 0.05, and interaction between COND and TEMPO, *F*(1.051,6.304) = 1.372, *p* > 0.05.

### Standard Deviation of Continuous Relative Phase

**FIGURE 6:**
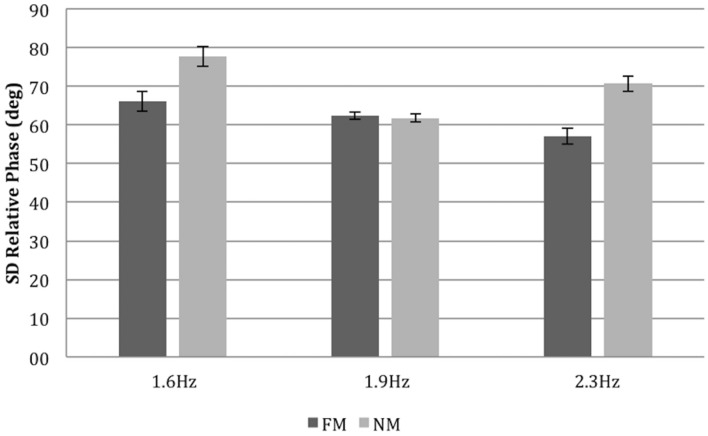
**Standard deviation relative phase as a function of COND (FM versus NM) and TEMPO (Hz).** Error bars denote between-subject (dyad) standard error.

For the standard deviation of the continuous relative phase, the ANOVA revealed a statistically significant effect of the mirror, i.e., COND, *F*(1,6) = 175.740, *p* < 0.001, $\eta_{p}^{2}$ = 0.967 showing that interpersonal coordination was on average more stable when one of the dancers faced the mirror (see **Figure 6**). The main effect of TEMPO was significant, *F*(2,12) = 10.868, *p* < 0.002, $\eta_{p}^{2}$ = 0.644, as was the interaction between COND and TEMPO significant, *F*(2,12) = 6.307, *p* < 0.013, $\eta_{p}^{2}$ = 0.512.

### Percentage of Time In-sync

For percentage of time of in-sync intervals, the ANOVA revealed no systematically induced longer sync-intervals as a consequence of the mirror, *F*(2,52) = 2.096, *p* > 0.05, $\eta_{p}^{2}$ = 0.412. The main effect for tempo revealed systematically induced longer sync-intervals, i.e., ranging from 46, 75–80% of the MT for the slow, medium and high tempo, respectively *F*(2,52) = 5.629, *p* < 0.01, $\eta_{p}^{2}$ = 0.839. Furthermore, off-line inspection of the raw data plots for re-synchronization during the trials, once the dyad had decoupled their synchronization, did not yield any results. Hence, once decoupled, the dyads remained so for the duration of the trial.

## Discussion

The aim of the current study was to characterize and investigate the effects of the use of the mirror on the interpersonal coordination in dance dyads. To this end we conducted kinematic analyses of one instructed (choreographed) movement sequence. Specifically, we asked if mirror-mirroring enabled dancers to synchronously dance in different spatial positions while simultaneously locked to an internalized auditory beat. Our results reveal a small positive effect, stabilizing unison movement when either member observed him/herself and confederate in the mirror but no facilitating effect of the mirror on dancers’ temporal synchronization. To our knowledge, these results indicate a first with regard to mirror-mirroring as a potential tool in dance performance; however, there are studies, which may support our findings. As mentioned earlier, choreographed (pre-planned) dance seems to rely heavily on shared representation of action observed and action executed (Calvo-Merino, 2004; Cross et al., 2011). With regard to music and silence we find compelling evidence for spontaneous coordination, for both seeing and hearing the other person rock in a rocking chair when placed side by side, with and without music (Demos et al., 2012). While this study additionally included a visual and non-visual condition, a key difference here is that Demos et al. (2012) observed emergent coordination while the present study examined planned coordination in the form of prescribed dance movement. Moreover, if we embed these concepts into our current understanding of joint action and joint coordination, one might conclude that the dancers in this study simply co-represented their own as well as their dance partner’s shared goal (Sebanz et al., 2003, 2005). However, the co-representation studies mentioned above did not purpose to capture ecologically valid events and thus do not offer a succinct comparison. Unique here was not the introduction of novel constraints but the manipulation and fragmentation of a movement sequence normally available to dancers via their normal dance training. Thus the ecological use of the mirror, their shared position combined with the occlusion of a partially closed curtain prevented total visual perception. This point is key for if dancers did not share vision as a modus operandi they could not operationally synchronize. We postulate here a possible motive for these findings: namely the concept of common coding.

### Common Coding

A basic understanding of common coding suggests that perception and action exploit common mental representation (Prinz, 1997). An underlying feature here is that sensory and motor programs do not communicate directly, rather translation possibilities occur that unravel both in an action-perception cycle. This has been tested across domains and leads to the posit that action observation induces action simulation (Wolpert et al., 2003; Wilson and Knoblich, 2005). In action studies with preplanned movements this would suggest that matching perceived and performed actions, enables one’s confederate to apply predictive models in their motor system that accurately predict the upcoming actions of their partner, and in tandem joint action outcomes (Becchio et al., 2008; Knoblich et al., 2011). These studies support our findings that in the execution of preplanned movement phrases using the mirror, dancers become proficient at predicting their confederate’s action by observing their partner(s) – and themselves– in the mirrored reflection. When both partners perform the task precisely, their joint behavior seems as if the dyad accurately synchronized their performance but synching is an emergent property of error-free individual performance. This line of reasoning is plausible in the presence of visual feedback. However, our participants’ completed near faultless symmetry in the absence of mirror-mirroring. Studies report that normally in the absence of visual feedback a proprioceptive drift is likely to occur (Wolpert et al., 1998), but not so quick as to degrade task integrity (Brown et al., 2003). A moderate drift was also observed in the present study as reflected by the variations in the percentage of movement time until out-of-sync. This could indicate distinct salient and decay-resistant coding of postures when mirror-mirroring and a less robust coding of movements when facing back to back (Rosenbaum et al., 1999).

### Tempo

As regards tempo the results diverged for the mirror and no-mirror conditions. When being able to see oneself and the confederate partially in a mirror, the medium and higher tempo slightly facilitated synchronization as well as coordination stability. In the absence of vision of oneself and partner via a mirror, the medium tempo proved the pace which yielded the strongest correlation between the dancer’s movements.

## Conclusions

The present study, even with only a modest seven dance dyads, shows that a fragmented view on one’s dance partner’s movements through a mirror, may aid in stabilizing *reflectionally* symmetric dance phrases, but not in a systematic manner. This null result could especially be of interest in dance education since dancers and dance trainers may think that the mirror might help synchronizing one’s dance with that of a partner, but overly convincing evidence for that reasoning was not confirmed in our study. Overall, the results support the hypothesis that when dancing together using an internalized beat, the use of a mirror provides an ecological means to stabilize interpersonal coordination in dance duets without an effect on synchronization.

## Author Contributions

DB has made a substantial contribution to conception design, acquisition of data, and analysis and interpretation of data. In addition DB initiated and participated in the drafting and revision of the article. RM has made a substantial contribution to conception design, analysis and interpretation of data. In addition RM participated in the drafting and revision of the article. DB and RM both have provided final approval of this manuscript provided here.

## Conflict of Interest Statement

The authors declare that the research was conducted in the absence of any commercial or financial relationships that could be construed as a potential conflict of interest.
